# Stress and reproductive events detected in North Atlantic right whale blubber using a simplified hormone extraction protocol

**DOI:** 10.1093/conphys/coaa133

**Published:** 2021-01-12

**Authors:** Katherine M Graham, Elizabeth A Burgess, Rosalind M Rolland

**Affiliations:** Anderson Cabot Center for Ocean Life at the New England Aquarium, Central Wharf, Boston, MA 02110, USA

**Keywords:** Anthropogenic impact, blubber hormones, entanglement, North Atlantic right whale, steroid hormone extraction, validation

## Abstract

As studies quantifying steroid hormones in marine mammal blubber progress, methodological refinements may improve the utility and consistency of blubber hormone measurements. This study advances blubber extraction methodologies by testing a simplified extraction protocol that reduces time and complexity compared to a protocol widely used in cetacean blubber studies. Using blubber samples archived from remote biopsy (*n* = 21 live whales) and necropsy collection (*n* = 7 dead whales) of North Atlantic right whales (NARW; *Eubalaena glacialis*) of known life history states, we performed analytical and biological validations to assess the feasibility of measuring reproductive (testosterone, progesterone) and glucocorticoid (cortisol) hormones in blubber via enzyme immunoassay following the simplified extraction. Analytical validations (parallelism, accuracy, extraction efficiency, repeatability) showed the simplified extraction produced similar results to the extended protocol, offering a more efficient and consistent technique. In live, apparently healthy whales, blubber testosterone concentrations (mean ± SE) were significantly higher in males (2.02 ± 0.36 ng/g) compared to females (0.81 ± 0.15 ng/g). Blubber progesterone was highest in a confirmed pregnant female (60.3 ng/g), which was 12-fold greater than the mean concentration of non-pregnant females (4.56 ± 0.88 ng/g). Blubber cortisol concentrations in whales that died from anthropogenic causes averaged 5.31 ± 2.28 ng/g, whereas most live, healthy whales had cortisol values below 1 ng/g. Among living whales, a whale actively entangled in fishing gear had the highest blubber cortisol measurement (3.51 ng/g), exhibiting levels similar to whales that died from acute entanglement (2.88 ± 0.42 ng/g). Overall, the highest blubber cortisol concentration (18.0 ng/g) was measured in a dead whale with a severe chronic entanglement, approximately 30-fold greater than mean blubber cortisol of apparently healthy whales (0.58 ± 0.11 ng/g). The methodological approach presented here provides a reference for researchers interested in an alternative, streamlined technique for hormone extraction of cetacean blubber and contributes to the diverse tool set for stress and reproductive assessments of endangered NARWs.

## Teaser

Analytical and biological validations of blubber hormone measurements were conducted for North Atlantic right whales. A simplified blubber hormone extraction protocol was successfully developed, improving the efficiency of sample preparation. Cortisol was elevated in whales that died from anthropogenic causes, and reproductive hormone concentrations showed meaningful patterns across known reproductive states.

## Introduction

Blubber has become a widely used sample matrix for reproductive and stress assessments of both odontocete (for examples see: Kellar *et al.*, 2006, Kellar *et al*., 2009, Trego *et al.*, 2013, Kellar *et al.*, 2015, Trana *et al.*, 2016, Champagne *et al.*, 2018), and mysticete whales (e.g. Mansour *et al.*, 2002, Kellar *et al.*, 2013, Vu *et al.*, 2015, Mello *et al.*, 2017, Pallin *et al.*, 2018, Carone *et al.*, 2019, Atkinson *et al.*, 2020). For instance, pregnant females can be readily identified using blubber progesterone concentrations in several whale species (Mansour *et al.*, 2002, Kellar *et al.*, 2013, Pallin *et al.*, 2018, Inoue *et al*., 2019, Atkinson *et al.*, 2020), and blubber cortisol measurements have shown promise for assessing human impacts (Kellar *et al.*, 2015) and environmental stressors (Trana *et al.*, 2016) on cetaceans. Blubber collected from free-swimming whales using remote biopsy methods or from dead whales during necropsy procedures can be used to explore physiological questions about specific individuals and populations (Hunt *et al.*, 2013, Rolland and Moore, 2018). Furthermore, the acquisition of blubber from numerous cetaceans has routinely occurred for other studies focusing on genetic or contaminant analysis (Noren and Mocklin, 2012, Booth *et al.*, 2020), with archived collections from previous efforts potentially available for hormone analysis (e.g. Trego *et al.*, 2013, Boggs *et al.*, 2019, Cates *et al.*, 2019).

Most blubber hormone studies have utilized immunoassays for quantification. In preparation for immunoassay, hormones are extracted from blubber tissue using an organic solvent. Nearly all published blubber hormone studies using immunoassays follow an extraction method outlined by Kellar *et al*. (2006, 2015), which was originally modified from Mansour *et al*. (2002). Although successful for tested species, this methodology is relatively complex, consisting of repeated solvent and supernatant transfers and requiring a variety of relatively hazardous chemicals (including diethyl ether, an extremely flammable chemical). Hormone extraction is the most labour-intensive component of sample analysis, and hence possibly the most error-prone part of the process because the margin of error increases with each additional step, which in turn could have consequences for data interpretation (Palme, 2005, Palme *et al.*, 2013). In the field of wildlife endocrinology, methodologies for extracting steroid hormones from many alternative sample matrices have been expanded and optimized over time (Wasser *et al.*, 2000, Palme, 2005, Hunt *et al.*, 2014, Burgess *et al.*, 2016, Hunt *et al.*, 2017, Richard *et al.*, 2017, Rolland *et al.*, 2019). Exploring simplification of complex extraction protocols, in tandem with validation testing of the procedure and resultant data, can help advance physiologic studies of wildlife populations (Palme *et al.*, 2013, Palme, 2019). Thus, it would be advantageous to develop a more streamlined hormone extraction protocol for cetacean blubber.

Endocrine studies using blubber tissue require careful biological validation and interpretation because blubber hormone measurements could be affected by sample collection (e.g. sampling depth, sample mass, specimen condition) and/or intrinsic factors (such as body condition or metabolism) (Kellar *et al.*, 2009, Kellar *et al.*, 2006, Kellar *et al.*, 2015, Trana *et al.*, 2015, Mello *et al*., 2017, Pettis *et al*., 2017). Many of the factors involved in the collection of blubber from cetaceans are inherently variable and not under the full control of researchers due to the logistics of remotely darting a free-swimming animal (e.g. the mass of blubber collected is influenced by the angle at which the dart strikes the whale) (Noren and Mocklin, 2012), or accessibility of carcasses (most whale caracasses beach in a state of advanced decomposition) (Mello *et al*., 2017). Given these circumstances, evaluation of hormone measurements can be strengthened by studying well-known individuals and populations. As demonstrated in a number of studies, the critically endangered North Atlantic right whale (NARW; *Eubalaena glacialis*) is a model species that has provided physiological validation of hormone analyses in alternative matrices (e.g. faeces, baleen, respiratory vapor) (Rolland *et al.*, 2005, Hunt *et al.*, 2016, Burgess *et al.*, 2018). This large whale species has been consistently monitored since 1980, and the North Atlantic Right Whale Identification and Sightings Database (www.rwcatalog.neaq.org) holds comprehensive sighting and life history data for individually identifiable whales (Hamilton *et al.*, 2007). Additionally, long-term assessment of faecal hormones in right whales have yielded extensive data on the endocrine patterns expected for various reproductive states in this species (Rolland *et al.*, 2005, Hunt *et al.*, 2006, Burgess *et al.*, 2017, Rolland *et al.*, 2017). Because NARWs face increased anthropogenic and environmental pressures (fishing gear entanglements, vessel interactions, human-generated underwater noise, climate change and shifting prey distributions) and non-sustainable reproductive rates (Meyer-Gutbrod and Greene, 2017, Corkeron *et al.*, 2018, Sharp *et al.*, 2019), the availability of efficient and diverse tool sets to monitor stress and reproduction is critical to guiding management and recovery efforts (Harcourt *et al.*, 2019).

The objectives of this study were to analytically validate a simplified protocol for extracting steroid hormones from blubber tissue and then, utilizing this simplified extraction method, characterize reproductive and stress-related hormones in blubber of live and dead NARWs of known life history states. To this end, we (i) conducted immunoassay validations to determine the feasibility of measuring three steroid hormone types (testosterone, progesterone and cortisol) in blubber of NARWs; (ii) evaluated a simplified blubber hormone extraction method alongside a more complex extraction protocol that is widely used in cetacean blubber studies; (iii) compared hormone concentrations in matched blubber and faecal samples to preliminarily examine concordance of blubber hormone measurements relative to a well-studied sample matrix for NARWs; (iv) examined blubber hormone profiles in apparently healthy, free-swimming NARWs of known sex and reproductive states; and (v) investigated blubber cortisol concentrations in whales that died from anthropogenic causes of entanglement in fishing gear and vessel strikes versus living whales.

## Materials and methods

### Sample collection

A total of 28 blubber samples archived from remote biopsy or necropsy of individual NARWs were used in this study. All samples were collected under federal permits to the New England Aquarium (NEAq) and Canadian Whale Institute (National Marine Fisheries Service permits: 655-1652, 655-1652-01, 14 233 and 19 674; Canada’s Department of Fisheries and Oceans permits under the Species at Risk Act) and the International Fund for Animal Welfare (National Marine Fisheries Service permits: 18786 and 18 786-02) and approved by NEAq’s Institutional Animal Care and Use Committee. Blubber biopsy samples (*n* = 21) were collected from free-swimming NARWs in the Bay of Fundy, Canada, from July through September in 2006–2017. Biopsy sampling was conducted using an Excalibur crossbow with 150-pound draw weight fitted with a custom made, floating dart containing a stainless steel collection tip of 7 mm diameter by 3 cm length (Brown *et al.*, 1991). The dart was aimed at the dorsal lateral region of the whale to remove a small plug of epidermis and underlying blubber (ranging in depth from 0.2 to 1.7 cm; mean 0.8 ± 0.43 cm). The biopsy sample was retrieved, and the epidermal layer was removed for genetic analysis (Frasier *et al.*, 2006). The remaining dermis and hypodermis (referred to as blubber) was archived for hormone analysis.

Blubber tissue sections (~10 x 10 x 10 cm) were dissected from dead whales (*n* = 7) during necropsies conducted in the months of April, May and August–October in 2016–2018 following standard necropsy procedures for NARWs (McLellan *et al.*, 2004; Sharp *et al*., 2019). The state of carcass decomposition was graded based on Geraci and Lounsbury (2005; see Table 1). If present, faeces were collected from the rectum during necropsy. Matched blubber and faecal samples were available from three whales, enabling comparison of hormone concentrations across matrices. All samples were kept frozen at −20°C or − 80°C until hormone analysis.

**Table 1:** Life history details of individual whales that were sampled for blubber in this study (total *n* = 28) using either remote biopsy (live whales) or necropsy procedures (dead whales).

Biopsy samples (*n* = 21)		
*Age class and sex*	*Number of individuals*	*Notes*
Adult females	5	pregnant, *n* = 1; lactating, *n* = 3; resting, *n* = 1
Juvenile females	4	active entanglement (moderate severity), *n* = 1
Adult males	7	
Juvenile males	5	
Necropsy samples (*n* = 7)		
*Age class and sex*	*Case number^(^* ^#^ *^)^*	*Cause of death*
Adult female	MME16–249 ^(3)^	Chronic entanglement
Adult female	IFAW18–281 ^(4)^	Acute entanglement
Juvenile female	IFAW17–182 ^(3)^	Blunt force trauma, vessel strike
Juvenile male	IFAW17–320 ^(4)^	Acute entanglement
Juvenile male	IFAW17–375 ^(4)^	Acute entanglement
Juvenile male	IFAW18–244 ^(3)^	Acute entanglement
Calf male	IFAW16–082 ^(3)^	Propeller trauma, vessel strike

# ^#^The decomposition code (graded from 2–5) assigned to the carcass at time of necropsy, as described by Geraci and Lounsbury (2005). Code 3: decomposed, but with organs intact. Code 4: severe decomposition, organs not recognizable, but carcass intact. Cause of death is the underlying condition that started the chain of events leading to death; from Sharp *et al*. (2019).

Individual whales were photographed and identified based on unique patterns of cornified epithelium (i.e. callosities) and permanent scars using the North Atlantic Right Whale Identification Database (Kraus *et al.*, 1986, Hamilton *et al.*, 2007, Right Whale Consortium, 2019), as well as genetic profiling of epithelial DNA (Frasier *et al.*, 2006, Frasier *et al.*, 2013, Right Whale Consortium, 2019). Whales were categorized based on age and reproductive history (Table 1; Hamilton *et al.*, 1998): calves (<1 year old, associated with their mother, likely nursing), juveniles (never calved and 1–8 y.o.), adults (year before first calving or ≥ 9 y.o.). Pregnancy was confirmed by identification of the female with a newborn calf in the year following sampling. Females sighted with a dependent calf at time of sampling were considered lactating. Adult females that were not pregnant or lactating were referred to as ‘resting’ (Rolland *et al.*, 2005). Biopsied whales were free-swimming and considered apparently healthy at sampling, except for one juvenile female (Eg4510) that was entangled in snow crab fishing gear at the time of sample collection. This whale was observed with a buoy and line exiting the left side of the mouth, and line exiting the right side of the mouth which was being pulled downward below the surface by the heavy weight of the gear. The whale had extensive rope abrasions across wide regions of the body and active bleeding at the peduncle region. Based on these observations, the entanglement injury was classified as moderate (defined as extensive skin abrasions or cuts that extended into the blubber; Knowlton *et al.*, 2015). These factors suggest that this whale had recently (within the last month) become entangled (Right Whale Consortium, 2019).

For necropsy cases, each dead whale was given a case number (Table 1). Two of the dead NARWs could not be assigned an individual identification due to decomposition of carcass; however, for both whales, sex was determined by visual observation or genetic analysis and age class (calf, juvenile or adult) was based on body length (Moore *et al.*, 2004). Cause of death was attributed to acute entanglement (hours to days) in four cases, chronic entanglement (weeks to months) in one case and blunt force and/or propeller trauma from vessel strike in two cases (Table 1). Further details on pathology and cause of death of these whales are described in Sharp *et al.* (2019).

### Hormone extraction

Blubber samples were trimmed of any remaining epidermal tissue using a clean scalpel blade. For all samples, 0.1 ± 0.05 g of blubber tissue was extracted. Sample masses of 0.1 g to 0.2 g have been widely used in blubber hormone studies; here, we chose to test protocols using the lower mass due to restricted amounts of tissue from biopsy collection. For biopsy samples less than 0.1 g, the entire blubber plug was extracted and only samples greater than 0.07 g were included in the study. For necropsy specimens, blubber was subsampled at a similar mass (0.1 g) and depth below the epidermis as biopsy samples to increase comparability between both sources of tissue collection.

Two different protocols for extracting hormones from blubber were tested: (1) an ‘extended’ protocol following methods described by Kellar *et al.* (2006, 2015), which was a modification of Mansour *et al*. (2002); and (2) a ‘simplified’ protocol adapted from a steroid tissue extraction protocol by immunoassay manufacturer, Arbor Assays (Ann Arbor, MI); see https://www.arborassays.com/assets/Tissue-Extraction-190402.pdf), with slight modifications to accommodate our laboratory equipment and reduce reagent volumes for a smaller sample mass.

Extended protocol: Full details are described in Kellar *et al.* (2015). In brief, this was a multi-step organic extraction consisting of homogenizing blubber (~0.1 g) in 1.0 ml of 100% ethanol (ACS reagent grade ≥ 99.5%; #459844, Sigma Aldrich) using an Omni Bead Ruptor 4 (catalogue #25–010, Omni International), followed by another wash step of 0.5 ml of ethanol. Resulting supernatants were collected, combined and evaporated and the residue resuspended in 2.0 ml of ethanol:acetone mix (4:1). The supernatant was transferred and evaporated before further extraction with 2.0 ml of diethyl ether. The supernatant was again collected and evaporated, then resuspended in 1.5 ml of acetonitrile (#271004, Sigma Aldrich) followed by the addition of 1.5 ml of hexane (#34859, Sigma Aldrich). The acetonitrile portion was separated, and an additional 1.5 ml of hexane added. The acetonitrile portion was again transferred, evaporated, and the final residue stored frozen at −20°C. Prior to immunoassay, sample extracts were resuspended in 0.5 ml of assay buffer (#X065, Arbor Assays) and vortexed thoroughly.

Simplified protocol: Blubber tissue (~0.1 g) was placed into homogenization tubes with grinding media (2.8 mm ceramic beads (catalogue #19–628) and one 6.5 mm ceramic bead (#19–682; Omni International)) and 1.0 ml of 100% ethanol. The sample was homogenized for six 45 s intervals using an Omni Bead Ruptor 4, similar to the extended protocol. The homogenate-ethanol mixture was transferred to a glass test tube (T1). The original homogenization tube with remaining grinding media was rinsed with 1.0 ml ethanol, vortexed and the supernatant was transferred to T1. Fluid in T1 was evaporated under airflow. Next, 2.0 ml of acetonitrile was added to the homogenate residue in T1, and the tube was vortexed (10 min) and then centrifuged (3500 rpm for 10 min at 4°C). The supernatant was transferred to a new tube (T2) followed by the addition of 4.0 ml of hexane, and the contents vortexed (5 min) then centrifuged to separate the acetonitrile and hexane layers. The acetonitrile layer was aspirated, transferred into a final tube (T3) and evaporated under airflow. Final dried extract residues were capped, sealed with parafilm and stored frozen (−20°C). Prior to immunoassay, sample extracts were resuspended in a mixture of 0.1 ml ethanol and 0.4 ml assay buffer (#X065, Arbor Assays) then vortexed thoroughly (2 min). The sample was allowed to rest at room temperature for 5 min before repeating the vortex and rest intervals twice more to solubilize the hormone.

### Hormone analysis

Immunoreactive testosterone, progesterone and cortisol were quantified in blubber extracts using commercially available enzyme immunoassay systems (catalogue #ISWE001, ISWE003, ISWE002, respectively; Arbor Assays, Ann Arbor, MI), following the manufacturer’s protocols. These bulk-reagent immunoassay kits were developed specifically for measuring hormones and their metabolites in alternative sample matrices from diverse wildlife species. All samples, standards and controls were assayed in duplicate, with the coefficient of variation (CV%) between all duplicates < 10%. Quality control samples of high (~30%) and low (~70%) binding were included on each plate, with resulting inter-assay CVs of 1.6% and 3.6% for testosterone (*n* = 7 assays); 6.2% and 11.4% for progesterone (*n* = 7 assays); and 1.8% and 5.3% for cortisol (*n* = 7 assays). Final results were reported as nanograms of immunoreactive hormone and metabolites per gram of blubber tissue (ng/g), subsequently referred to simply as blubber testosterone, progesterone and cortisol. Antibody cross-reactivity, assay sensitivity and lower limit of detection values are available on the manufacturer’s website: www.arborassays.com/products/.

### Analytical validations

Blubber from dead whales was used to conduct analytical validations and evaluate both hormone extraction methodologies. These large sections of blubber tissue could be repeatedly subsampled and provided matched pairs of near-identical samples from the same localized region of blubber tissue enabling comparison of extraction techniques.

First, to ensure the selected immunoassays could reliably detect and measure the three hormones of interest in NARW blubber extracts, we conducted the following analytical validations: (i) parallelism; and (ii) accuracy. Parallelism was tested by serially diluting a pool of blubber extracts (from 1:1 (neat) to 1:256) and assessing the resulting dilution curve against the standard curve for differences in slope. Expected results should show no significant difference between the curves (F-test, *P* > 0.05), indicating the assay can reliably detect the hormone of interest (Grotjan and Keel, 1996). Assay accuracy was tested by spiking the standard curve with an equal volume of pooled sample extracts. When plotted, observed versus expected hormone values should be linear (ideal *r*^2^ > 0.95) with a slope between 0.7–1.3 (ideal slope = 1.0), demonstrating that the sample matrix does not interfere with antibody binding (Ezan and Grassi, 2000, Grotjan and Keel, 1996).

Next, to evaluate the suitability of using a simplified extraction protocol as an alternative to the widely used extended extraction, we conducted experimental comparisons using both extractions protocols based on: (iii) extraction efficiency; (iv) within-extraction method variation; and (v) comparison of final hormone measurements. Extraction efficiency was tested using a separate set of 10 biopsy-sized blubber subsamples (~0.1 g each) for each individual hormone of interest. Six of these subsamples were placed into individual homogenization tubes and each tube was spiked with a known concentration of hormone at 40 ng in dH_2_O, and then left overnight at 4°C to allow the hormone solution to soak into the blubber. The other four subsamples were each placed into homogenization tubes containing dH_2_O without added hormone (non-spiked) and left overnight at 4°C. The following day, subsamples were assigned to either the simplified extraction or the extended protocol (*n* = 3 spiked and *n* = 2 non-spiked for the two protocols; *n* = 10 total for each hormone) before immunoassay. Extraction efficiency (%) was calculated as the mean concentration of hormone minus mean background (non-spiked samples), divided by the known amount of hormone added before extraction and multiplied by 100 (Palme, 2019). Within-extraction method variation tested the precision or repeatability of a hormone measurement across multiple extracts generated by each extraction protocol. For this test, blubber was dissected into 20 subsamples that were randomly assigned to extraction using either the simplified protocol (*n* = 10) or extended protocol (*n* = 10). Within-extraction method variation was quantified as the CV% between hormone measurements of 10 replicate extracts per protocol. Finally, we assessed the differences in absolute hormone concentration measured in paired subsamples taken from each of the seven dead whales. For each whale, four blubber subsamples were taken, which allowed for two subsamples to be assigned to each extraction protocol. The resultant extracts were assayed, and hormone concentrations were averaged for each extraction method, with the final measurements compared between the two methods.

To examine concordance of blubber hormone concentrations to faeces (a previously validated and well-studied sample matrix for measuring hormones in NARWs), we used matched blubber and faecal samples that were collected from three dead whales. Faecal samples were processed and analysed for faecal androgens, progestagens and glucocorticoids following methods described by Rolland *et al.* (2005) and Hunt *et al.* (2006). Blubber samples were extracted by the simplified extraction prior to measurement.

### Blubber hormone concentrations of NARWs and biological validation

To characterize reproductive and glucocorticoid hormone concentrations in NARW blubber, testosterone, progesterone and cortisol were measured using the validated Arbor Assays immunoassay systems (see *Analytical validations*). Based on validation results, blubber samples from all whales (*n* = 28) were extracted using the simplified extraction protocol and resulting sample extracts were diluted 1:3 in assay buffer (#X065, Arbor Assays) prior to assay. Hormone data were compared across whales of different sexes, age classes, reproductive states and health statuses to evaluate whether blubber sample measurements reflect endocrine profiles expected for whales of known life history states.

### Data analysis

Data from analytical validation tests of parallelism, accuracy, extraction efficiency and within-extraction method variation were compared between extraction protocols. A paired *t-*test was used to assess differences in measured hormone concentrations of blubber subsamples extracted following each protocol. Hormone values in matched blubber and faecal samples for three individuals, were graphically presented to observe congruence of trends between these alternative matrices. Descriptive statistics (mean ± SEM) were used to summarize the data set. Hormone concentration data were log_10_-transformed for the following analyses to meet assumptions of normality and homogeneity of variance, which were tested using Shapiro–Wilk test and Levene’s test. Blubber hormone concentrations of live, apparently healthy whales (*n* = 20 out of 21 biopsy samples; one biopsy (from Eg4510) was excluded due to active entanglement) were examined using a univariate general linear model (GLM). A full factorial model was used to analyse the effect of sex, age class (i.e. juvenile or adult) and their interaction on hormone concentrations (dependent variable) of whale blubber samples, with the following equation: *y_i_* = β_0_ + β_1_ sex*_i_* + β_2_ age class *_i_* + β_3_ sex × age class*_i_* + ϵ*_i_* where *y* is the response variable, β is the population slope and fixed effect parameters (including β_0_ as the population intercept) and ϵ is a random error term associated with the *i*th observation. To avoid omission of any individual whale due to missing data fields in the GLM, we deliberately classed one female of uncertain age as ‘adult’. This decision was grounded on available data that showed this female was older than 7 years of age (based on sighting records) and successfully calved 17 months after sampling—and therefore, this female was presumed to be nearing reproductive maturity when blubber sampling occurred.

To consider the possible effect of abiotic factors on measured hormone variables in the full set of blubber samples (*n* = 28; both live and dead whales), we used a multivariate GLM. Key attributes of sample storage time (i.e. number of years from sample collection until hormone analysis), mass of the analysed sample (measured in grams) and whale survival at time of sampling (i.e. live or dead whale, as associated with biopsy or necropsy sampling) were included as explanatory variables into the model designed to analyse all dependent variables (testosterone, progesterone and cortisol concentrations) simultaneously, with the following equation: *z_ik_* = constant + *c*_1_ storage time*_i_* + *c*_2_ sample mass*_i_* + *c*_3_ whale surivival*_i_* where *z* is the combination of response variables (observation *i* for the linear combination *k)* and *c* is the coefficient measuring the relative contribution of each variable. Univariate between-subjects F-tests that indicated the effect of each factor on each dependent variable were also produced by the GLM framework. All statistical analyses were performed using SPSS (version 25) and significance level was set at 0.05 for all statistical tests.

## Results

### Analytical validations

**Figure 1:**
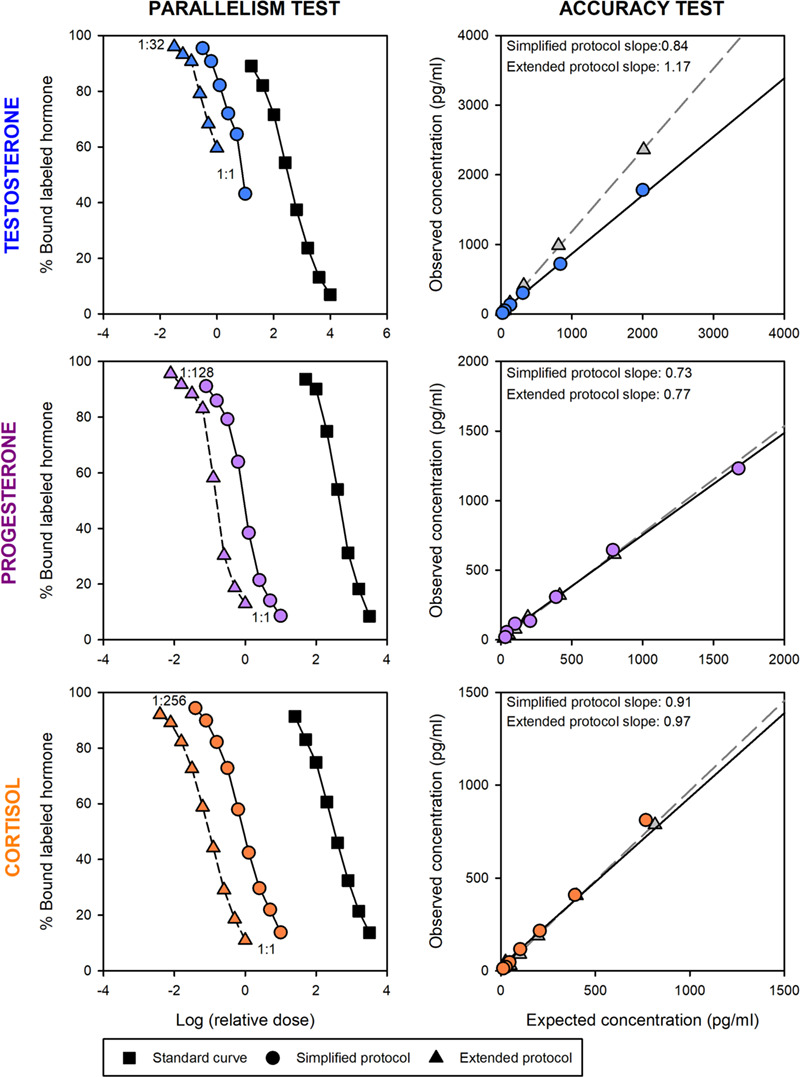
Validation test plots for testosterone (top row), progesterone (middle) and cortisol (bottom) of NARW blubber extracts using the simplified extraction protocol (circles: ● with solid line) or extended extraction (triangles: ▲ with dashed line). Parallelism (left column) was observed between serially diluted sample curves (dilution range reported for each hormone) and standard curves (squares: ■) for both extraction methods across all hormones. [Note: In parallelism graphs, the relative dose (x-axis) of the sample serial dilution curves was displaced to avoid overlap]. Assay accuracy (right column) was demonstrated by the positive linear relationship of expected hormone concentration against observed concentration in spiked samples (simplified extraction protocol: circles ●; extended extraction: triangles▲) and regression line slopes within the acceptable range of 0.7–1.3 (exact value reported on each graph).

For both extraction protocols, serially diluted blubber extracts demonstrated parallelism to the standard curve for testosterone, progesterone and cortisol immunoassays (all *P* > 0.05; Fig. 1 and Table 2). Blubber extracts derived using the simplified method demonstrated reasonable accuracy for all hormones tested and yielded similar results to the extended protocol (all slopes between 0.7 and 1.2, *r^2^* > 0.99; Fig. 1 and Table 2). Analytical validation results indicated that hormone metabolites extracted from NARW blubber by either protocol can be detected by the assay antibody across a range of concentrations (parallelism test) and that substances inherent to the extract matrices do not interfere with accurate hormone measurement (accuracy test).

**Table 2:** Analytical validation results (parallelism, accuracy, extraction efficiency and within-extraction method variation) for the simplified and extended extraction protocols.

	Parallelism (F-test_(df)_; *P*-value)		Accuracy test (linear slope)	
Hormone	Simplified extraction	Extended extraction	Simplified extraction	Extended extraction
Testosterone	*F*_(1,9)_ = 1.23; *P* = 0.30	*F*_(1,8)_ = 0.18; *P* = 0.68	y = 0.84x + 26.85	y = 1.17x + 15.35
Progesterone	*F*_(1,10)_ = 0.65; *P* = 0.44	*F*_(1,9)_ = 0.37; *P* = 0.56	y = 0.73x + 18.70	y = 0.77x—0.43
Cortisol	*F*_(1,12)_ = 0.08; *P* = 0.79	*F*_(1,12)_ = 0.09; *P* = 0.76	y = 0.91x + 24.88	y = 0.97x + 0.88
	Extraction efficiency (% recovery)		Within-extraction method variation (mean %CV)	
Hormone	Simplified extraction	Extended extraction	Simplified extraction	Extended extraction
Testosterone	74%	81%	11.4%	21.6%
Progesterone	70%	55%	14.9%	35.2%
Cortisol	61%	67%	6.4%	19.1%

Extraction efficiency ranged from 61–74% for the simplified protocol and 55–81% for the extended protocol (Table 2). Both protocols had similar overall recoveries across all hormones (mean 68% for both protocols). The simplified protocol resulted in higher recovery of progesterone (70% compared to 55%), but conversely, slightly higher recovery of testosterone was observed for the extended protocol (81% compared to 74%). For cortisol, relatively similar extraction efficiencies were found for both the simplified (61%) and extended extraction protocols (67%). Within-extraction method variability was lower for samples extracted by the simplified protocol (range 6.4–14.9%) compared to the extended method (19.1–35.2%), with the best result for the measurement of cortisol (6.4%) (Table 2).

**Figure 2:**
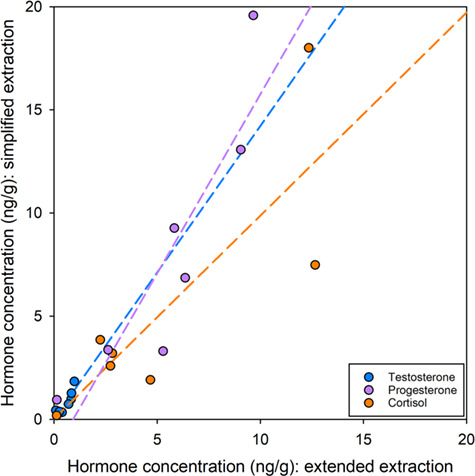
Comparison of hormone measurements (ng/g) in matched blubber subsamples extracted using the extended (x-axis) and simplified (y-axis) protocols. Coloured dotted lines represent the linear regression equation for each hormone type; testosterone (blue): y = 1.42x + 0.02; progesterone (purple): y = 1.74x – 1.60; cortisol (orange): y = 0.98x + 0.03.

Overall, hormone measurements from matched subsamples extracted using the two protocols were similar for all hormone types (testosterone: *t*_(6)_ = −2.01, *P* = 0.09; progesterone: *t*_(6)_ = −1.69, *P* = 0.14; cortisol: *t*_(6)_ = 0.31, *P* = 0.98). Generally, testosterone and progesterone concentrations were higher in extracts generated using the simplified extraction protocol (averaging 1.9 and 2.2 times higher, respectively) compared to the extended extraction protocol extracts (Fig. 2). At higher sample concentrations, there was greater variation in resulting hormone values between the simplified and extended protocols (Fig. 2).

**Figure 3:**
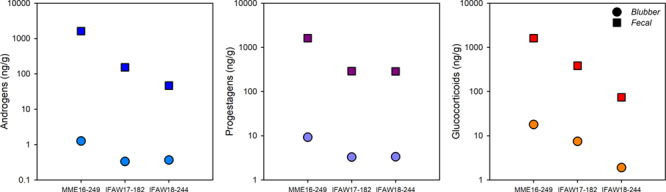
Matched blubber (circle) and faecal (square) hormone values (ng/g) collected from three whales during necropsy procedures. Patterns in blubber hormones showed similar trends to faecal hormones, albeit at concentrations two or three orders of magnitudes lower than in faeces. The y-axis is presented as a log scale.

Blubber hormone concentrations paralleled faecal hormone patterns for three whales with matched sample types, with hormone concentrations in blubber two or three orders of magnitude lower than faeces (Fig. 3). In both blubber and faeces, the highest concentrations of reproductive hormones (testosterone and progesterone) were observed in the adult female (MME16–249) compared to two juvenile whales. The highest blubber and faecal glucocorticoid concentrations were also measured in whale MME16–249 that died following a severe, chronic entanglement (Table 1; Sharp *et al*., 2019).

### Blubber hormone concentrations of NARWs and biological validation

**Figure 4:**
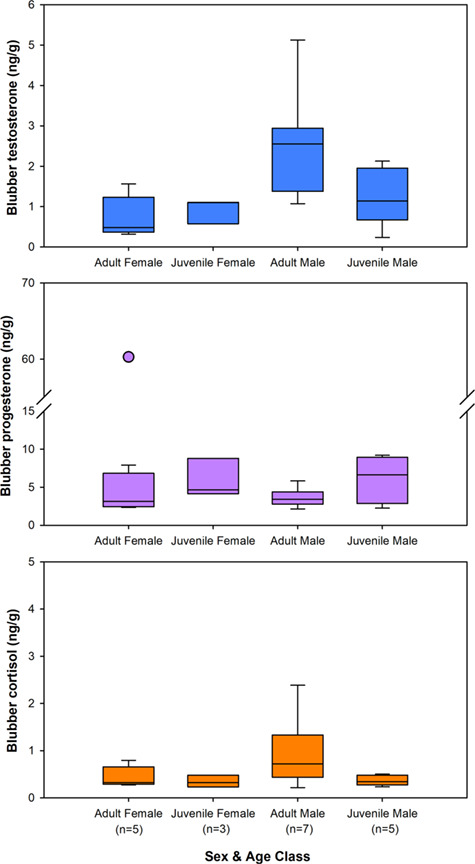
Blubber testosterone (top, blue), progesterone (middle, purple) and cortisol (bottom, orange) of live, apparently healthy NARWs across sexes and age classes. Boxplots encompass first and third quartiles, the line inside the box indicates the median value and whiskers represent the 10th and 90th percentiles. Note: In the progesterone graph, a break was inserted in the y-axis between 15 and 55 ng/g and the value for the pregnant female (denoted by a filled circle) was plotted separately due to its extremely high concentration.

Testosterone, progesterone and cortisol were measurable in all NARW blubber samples following extraction with the simplified protocol. In live, apparently healthy right whales, blubber testosterone concentrations were significantly higher in males (2.02 ± 0.36 ng/g; *n* = 12) than females (0.81 ± 0.15; *n* = 8) (*F*_1,16_ = 5.90, *P* = 0.03). Mean blubber testosterone of adult males (2.54 ± 0.50 ng/g; *n* = 7) was approximately twice as high as juvenile males (1.28 ± 0.32 ng/g; *n* = 5) and over three times greater than adult females (0.74 ± 0.17 ng/g; *n* = 5) (Fig. 4); however, differences associated with age class did not achieve statistical significance (*F*_1,16_ = 0.52, *P* = 0.48; interaction term: *F*_1,16_ = 3.7, *P* = 0.07). Blubber progesterone levels were similar in females (11.80 ± 0.12 ng/g; *n* = 8) and males (4.70 ± 0.01 ng/g; *n* = 12) (*F*_1,16_ = 0.74, *P* = 0.40), as well as across age classes (*F*_1,16_ = 0.11, *P* = 0.74; interaction term: *F*_1,16_ = 0.54, *P* = 0.47). However, the highest blubber progesterone concentration (60.30 ng/g) was measured in a confirmed pregnant female (Fig. 4). This value was over 12-fold greater than mean blubber progesterone of non-pregnant females (4.56 ± 0.88 ng/g; *n* = 7).

For live, apparently healthy whales in this study, blubber cortisol concentrations were not significantly different between sexes (*F*_1,16_ = 1.70, *P* = 0.21), age classes (*F*_1,16_ = 3.26, *P* = 0.09), or reproductive groups (interaction term: *F*_1,16_ = 0.97, *P* = 0.34). However, adult males had blubber cortisol levels (0.94 ± 0.27 ng/g; *n* = 7) that averaged two times higher compared to juvenile males (0.37 ± 0.05 ng/g; *n* = 5), juvenile females (0.34 ± 0.07 ng/g; *n* = 3) and adult females (0.44 ± 0.10 ng/g; *n* = 5) (Fig. 4).

In the analysis examining abiotic factors, we found that storage time (Pillai’s Trace = 0.24; *F*_3,22_ = 2.33, *P* = 0.10), sample mass (Pillai’s Trace = 0.09; *F*_3,22_ = 0.75, *P* = 0.53) and whale survival (Pillai’s Trace = 0.76; *F*_3,22_ = 2.31, *P* = 0.10) did not exhibit significant effects on measured hormone concentrations of blubber samples. Univariate tests also showed that storage time (7.7 ± 0.9 years; range: 0.4–12.6 years) and sample mass (0.10 ± 0.003 ng/g; range: 0.07–0.12 ng/g) did not significantly influence blubber testosterone, progesterone or cortisol measurements in this study (all *P* > 0.05). There was no effect of whale survival on reproductive hormone measurements, with similar blubber testosterone concentrations in live (1.48 ± 0.25 ng/g) and dead whales (0.85 ± 0.21 ng/g) (*F*_1,24_ = 0.69, *P* = 0.42), and similar levels of blubber progesterone in live (7.30 ± 2.70 ng/g) and dead whales (8.05 ± 2.47 ng/g) (*F*_1,24_ = 2.89, *P* = 0.10). However, there was a significant influence of whale survival on blubber cortisol concentrations (*F*_1,24_ = 6.90, *P* = 0.02).

**Figure 5:**
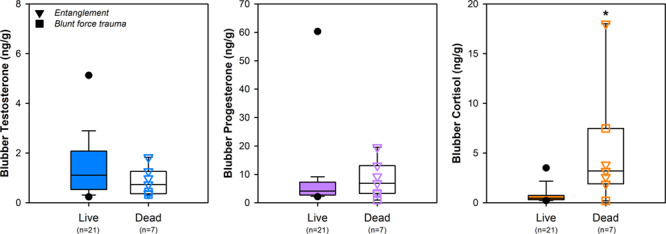
Blubber hormone concentrations for live whales (biopsy samples, *n* = 21) compared to necropsy samples from whales that died from anthropogenic causes (*n* = 7). For live whales, outliers beyond the 5th and 95th percentile are plotted with a circle. For dead whales, individual hormone values are plotted over the boxplot, with whales that died as a result of trauma from entanglement denoted by triangles (▼; *n* = 5) or vessel strike by squares (■; *n* = 2). Asterisk (*) indicates a significant difference between the live and dead whales at *P* < 0.05.

Blubber cortisol concentrations of whales that died from anthropogenic causes were significantly greater (5.31 ± 2.28 ng/g; *n* = 7) than living whales (0.72 ± 0.18; *n* = 21), which typically had levels below 1 ng/g (Fig. 5). Notably, however, one live whale had an extreme cortisol concentration (identified as an outlier, Fig. 5) and this individual whale (Eg4510) was actively entangled in fishing line at the time of biopsy collection; whereas, all other live whales were free-swimming and considered apparently healthy. Whale Eg4510 had recently acquired an entanglement (classified as moderate in severity) and her blubber cortisol concentration (3.51 ng/g) was comparable to levels measured in whales that died from acute entanglement (2.88 ± 0.42 ng/g; *n* = 4). One dead whale that sustained a severe, chronic entanglement (MME16–249) had the highest blubber cortisol concentration (18.01 ng/g) measured in this study. Of the two whales that died from injuries related to vessel strikes, one whale (IFAW16–082) that suffered propeller-induced trauma had the lowest measured cortisol value in the study of 0.19 ng/g, whereas the other whale (IFAW17–182) that suffered blunt force trauma had a relatively high cortisol concentration (7.30 ng/g).

## Discussion

This study presented a simplified protocol for extracting hormones from cetacean blubber and demonstrated the simplified extraction is a consistent and efficient alternative to a widely used extended protocol (Kellar *et al.*, 2006, 2015) for this essential sample preparation step. We performed and evaluated both extraction protocols (simplified and extended) to obtain comparable data on hormone measurement results for testosterone, progesterone and cortisol, providing a useful reference for future researchers. Moreover, data reported here are the first quantification of reproductive (testosterone and progesterone) and glucocorticoid (cortisol) hormones in NARW blubber tissue and revealed biologically meaningful hormone patterns can be measured in blubber, making it a valuable matrix for assessing reproductive and stress-related states in free-swimming whales, as well as for postmortem investigation.

### Analytical validations

The simplified extraction protocol increased the efficiency and reproducibility of blubber hormone measurements and proved to be a reliable extraction technique for cetacean blubber studies. Using the simplified protocol, sample processing time was substantially reduced (>50%) and required fewer steps and hazardous chemicals (i.e. removal of highly volatile diethyl ether), making this simplified blubber hormone extraction technique potentially feasible for laboratories with limited resources (e.g. protective equipment, labour and supply costs). Most importantly, precision of hormone measurements was shown to improve when using the simplified extraction protocol, as all intra-sample CV values for the simplified protocol were near the standards recommended for wildlife endocrinology (i.e. <10%; Grotjan and Keel, 1996), with the best result for the measurement of cortisol (CV of 6.4%). Hormone extraction should be kept as simple as possible because additional steps increase the extent of variation, which could potentially impact accuracy of the final measurement (Burd, 2010, Palme, 2019). We posit that the higher variation measured between replicate samples extracted using the extended protocol may have resulted from inconsistent losses in hormones during repeated supernatant transfer steps. Kellar *et al.* (2006) also found high variation between identical samples when reporting on the use of the extended protocol for progesterone measurement (CV of 18%) but concluded that high variability inherent to this extraction methodology did not impede pregnancy determination. Nonetheless, it is preferable to minimize sources of intra-sample variability, particularly when detecting physiological changes at lower hormone concentration ranges (Millspaugh and Washburn, 2004, Watson *et al.*, 2013). The high variability associated with the extended extraction could be problematic, particularly for stress assessments that may be used in conservation management decisions, clinical diagnostics, or developing endocrine reference ranges for populations.

Extraction efficiency for the two protocols varied across hormone types, however this variation is expected given the range of wash steps and reagents with varying polarities used in each extraction protocol. Furthermore, extraction efficiency calculations that are based on adding exogenous parent steroids to the sample prior to extraction are often considered an artificial measure of true recovery (Palme *et al.*, 2013, Palme, 2019), particularly when hormone metabolites predominate in the tissue, as is the case for blubber (Boggs *et al.*, 2017, Atkinson *et al.*, 2020). Nonetheless, this approach does hold value in the present study for comparing between different extraction protocols. Refinement of the simplified extraction protocol, such as modifying the polarity and/or types of solvents used, may improve extraction efficiency and recovery further.

Successful analytical validation of commercial enzyme immunoassays tested in this study establishes these assays as suitable for measuring testosterone, progesterone, cortisol and associated metabolites in NARW blubber. Concentrations of hormones in NARW blubber were generally low, but comparable to levels measured in blubber of other large whale species (Clark *et al.*, 2016, Cates *et al.*, 2019, Atkinson *et al.*, 2020, Mingramm *et al.*, 2020). Studies using LC–MS/MS have established that cetacean blubber contains parent steroids (cortisol, progesterone and testosterone) as well as their metabolites, including cortisone, 17-hydroxyprogesterone, 11-deoxycorticosterone, 11-deoxycortisol and androstenedione (Boggs *et al.*, 2017, Galligan *et al.*, 2018, Boggs *et al.*, 2019, Dalle Luche *et al.*, 2019). Analysis of blue whale blubber showed progesterone was present in extracts (5%), although the majority of screened fractions (~67%) were found to be a more polar progesterone metabolite (Atkinson *et al.*, 2020). The use of broad-spectrum antibodies in this study permitted the quantification of blubber metabolite concentrations in all samples from NARWs. Future LC–MS/MS analysis of NARW blubber could be used to identify the predominant steroids and metabolites present in this tissue, enabling selection of more targeted immunoassay systems that may offer additional physiological insights from blubber; for example, identifying different stages of pregnancy based on shifts in the predominant steroids and metabolites (Legacki *et al*., 2020).

### Blubber hormone concentrations of NARWs and biological validation

Blubber testosterone, progesterone and cortisol in live, apparently healthy right whales followed expected physiologic patterns based on sex and reproductive state and were consistent with well-established faecal hormone patterns for the species (Burgess *et al.*, 2017, Hunt *et al.*, 2006, Rolland *et al.*, 2005, Rolland *et al.*, 2017). Adult male NARWs had higher testosterone and cortisol (and/or metabolites) in blubber compared to non-pregnant females and immature animals, presumably related to reproductive activity in males (Rolland *et al.*, 2005, Hunt *et al.*, 2006, Rolland *et al.*, 2017). A confirmed pregnant female was distinguished from non-pregnant animals by extremely high blubber progesterone concentrations (12-fold increase). Such physiological changes associated with pregnancy have also been measured in NARW faeces (Rolland *et al.*, 2005) and in the blubber of other large whale species (Kellar *et al.*, 2013, Clark *et al.*, 2016, Pallin *et al.*, 2018, Goertz *et al.*, 2019, Atkinson *et al.*, 2020). Most adult females in our biopsy sample set were lactating, with only one non-pregnant female considered to be in a resting state. Increasing sample sizes for reproductive females will better delineate the range of progesterone concentrations associated with reproductive cycling and pregnancy in blubber tissue.

Matched faeces and blubber collected from three individuals provided evidence that hormone patterns were similarly reflected in both matrices, though at different quantitative scales. This finding is consistent with bowhead whale progesterone concentrations that showed concordance among blubber, urine and serum samples (Kellar *et al*., 2013). Since blood sampling and standard endocrine validations are not possible for most large whales, our preliminary data on matched alternative matrices lends further validity to the use of blubber hormone techniques for physiological assessment. Additionally, blubber hormone measurements may be useful to examine seasonality in free-swimming NARWs, a topic which remains understudied since faecal samples are often not obtainable during annual periods of fasting.

Abiotic factors (including storage time, sample mass and whale survival at time of sampling) should be considered when comparing different sources of tissue and these factors did not appear to hamper interpretation of hormone results in this study. We noted similarities between living and dead whales for both reproductive hormones (testosterone and progesterone), suggesting that carcasses in this study were still viable for hormone measurement. Furthermore, there was a wide variation in cortisol levels among dead whales (spanning the lowest and highest cortisol measurements in this study), suggesting these patterns were not due to tissue decomposition, and instead meaningfully reflect the time course of mortality or injury (similar to NARW faecal glucocorticoid patterns reported in Rolland *et al*., 2017).

Cortisol data suggest that adrenal activation due to stressful anthropogenic impacts was captured in blubber tissue and the mode by which an animal died (entanglement in fishing gear or vessel strike) was the primary driver of postmortem cortisol levels. Increased blubber cortisol concentrations have been reported in other cetaceans following stressful events, including beach stranding of short-beaked common dolphins (Kellar *et al.*, 2015) and humpback whales (Mingramm *et al*., 2020), and entrapment of beluga whales in sea ice (Trana *et al.*, 2016). The whale with the highest blubber cortisol measured in this study (MME16–249) died from a severe, chronic entanglement in fishing line that occluded the rostrum and was cinched at the flippers, restricting the ability of the mouth to open for feeding (Sharp *et al*., 2019) leading to a prolonged decline in health and likely heightened adrenal activation. By contrast, the lowest measure of cortisol came from a whale (IFAW16–082) that died from propeller-induced trauma involving a deep laceration into the abdominal cavity, vertebral shearing and skull fractures (Sharp *et al*., 2019). The trauma suffered by this individual likely led to a rapid death, with limited time for activating a stress response and/or uptake of hormone into blubber tissue, such that cortisol levels in the blubber of this whale reflected a prior physiological state of an otherwise apparently healthy individual preceding vessel strike. The other whale that sustained blunt force trauma from a vessel strike (IFAW17–182) had somewhat elevated cortisol levels but showed evidence of other pathologies that may have heightened adrenal activity in this individual prior to death (Sharp *et al*., 2019). All four dead whales that were classified as acute entanglement cases showed intermediate cortisol levels, with evidence that two of these whales drowned (potentially an acute death) as a result of their entrapment in fishing gear (Sharp *et al.*, 2019). Blubber levels are likely a function of total cortisol production, with a lag time before accumulating in this peripheral tissue (possibly on the order of weeks to months for large whales, based on progesterone signal dynamics in pregnant bowhead whales (Kellar *et al*., 2013)). Ultimately, using blubber glucocorticoid measurements for stress assessment in large whales may be most applicable for assessing threats sustained over longer period (weeks to months) rather than shorter term impacts (hours to days).

## Conclusions

This study presents a useful and practical contribution towards advancing blubber hormone assessments for marine mammal populations by developing and validating a reliable, simplified hormone extraction protocol and then applying it to evaluate blubber hormone concentrations in well-studied NARWs. Optimization of blubber hormone measurements has the potential to expand the reach and reliability of this approach, benefitting researchers and management agencies studying vulnerable marine mammal populations. Using a small mass of blubber, we were able to measure and compare three different hormone types for reproductive and stress assessment of a large whale. Many blubber studies using immunoassays have focused on measuring a single hormone. However, the capacity to examine a suite of hormones is valuable for interpreting physiologic patterns, particularly because factors such as reproductive state can influence other hormone measures (e.g. adrenal hormones) (Hunt *et al*., 2006, Sheriff *et al.*, 2011). Additional hormone types, such as thyroid hormones and aldosterone could also be explored in marine mammal blubber, as these data may be beneficial for more detailed physiological assessment. The hormone values reported here are important for establishing reference ranges of physiological information to which we can compare in future studies, especially given the increasing impact of human activities on the ocean (Maxwell *et al*., 2013, Fleishman *et al*., 2016) and animal welfare concerns (Moore and van der Hoop, 2012, Rolland *et al*., 2017, Papastavrou *et al*., 2017). Physiologic profiles measured in blubber are valuable for assessing the lethal and sublethal effects of major anthropogenic threats, including entanglements in fishing gear and vessel strikes, on NARWs as well as other vulnerable marine mammal populations.

## Funding

This work was supported by Fisheries and Oceans Canada: Ocean Ecology Section (contract # F5211–180767).
